# Psychiatric comorbidities in hoarding disorder: a structured clinical interview study

**DOI:** 10.3389/fpsyt.2026.1909322

**Published:** 2026-09-03

**Authors:** Nami Nishida, Keitaro Murayama, Hirofumi Tomiyama, Kenta Kato, Kang Mingi, Kou Matsukuma, Makoto Kawahito, Kana Tsunoda, Takuro Kamio, Ayaka Shuto, Tomohiro Nakao

**Affiliations:** 1 Department of Neuropsychiatry, Graduate School of Medical Sciences, Kyushu University, Fukuoka, Japan; 2 Kyushu University Hospital, Fukuoka, Japan

**Keywords:** attention-deficit/hyperactivity disorder (ADHD), hoarding disorder, major depressive disorder, obsessive-compulsive disorder (OCD), psychiatric comorbidities, structured clinical interview diagnosis

## Abstract

**Objective:**

Hoarding disorder (HD) is a psychiatric condition characterized by persistent difficulty in discarding possessions, leading to significant clutter and functional impairment. HD is often comorbid with other psychiatric disorders, but previous findings of these comorbidities are inconsistent due to limited structured assessments. This study investigates the psychiatric comorbidities in individuals with HD using structured, clinician-administered interviews.

**Methods:**

Between 2016 and 2022, 68 individuals diagnosed with HD were recruited from an outpatient clinic at Kyushu University Hospital. Comorbid psychiatric disorders were assessed using the Structured Clinical Interview for DSM, Conners’ Adult Attention-deficit/hyperactivity disorder (ADHD) Diagnostic Interview, and additional standardized scales. Demographic, clinical, and diagnostic data were collected.

**Results:**

Eighty percent of the participants had at least one comorbid psychiatric disorder. ADHD was the most common comorbid disorder, occurring in 29.4% of the participants (95% confidence interval [CI], 18.6–40.3), followed by obsessive-compulsive disorder in 26.5% (95% CI, 16.0–36.9) and major depressive disorder in 20.6% (95% CI, 11.2–30.7). Among the participants with ADHD, 55% had the predominantly inattentive presentation and 45% had the combined presentation; none had the predominantly hyperactive-impulsive presentation.

**Conclusion:**

The most common comorbidity associated with HD is ADHD. The significance of this study lies in the fact that a structured interview with a clinician was also used to diagnose ADHD; we believe that identifying comorbidities in HD will allow future research to better understand the related clinical features, neuropsychological testing, and imaging studies.

## 1 Introduction

Hoarding disorder (HD) is characterized by a persistent difficulty in discarding or parting with possessions, regardless of their actual value, resulting in clutter that compromises the use of living spaces and causes significant social and functional impairment. HD commonly co-occurs with various psychiatric disorders (1). Among these, major depressive disorder (MDD) is the most commonly observed comorbidity (1–3). In addition, attentional deficits have been shown to significantly predict hoarding severity (4), and hoarding symptoms are frequently reported in individuals with attention-deficit/hyperactivity disorder (ADHD), suggesting a notable overlap between these two conditions (5–8). Recent evidence has further strengthened interest in the relationship between ADHD and HD. A recent study reported that adults with ADHD who exhibited hoarding symptoms showed more complex clinical and cognitive characteristics than adults with ADHD without such symptoms (9).

Psychiatric comorbidities may influence the core symptoms of HD, including excessive acquisition, difficulty discarding, and clutter. These comorbidities may also affect the cognitive underpinnings of hoarding, such as beliefs about value, utility, responsibility, and the potential loss associated with possessions (10). For example, a heightened sense of responsibility commonly observed in patients with obsessive-compulsive disorder (OCD) may exacerbate difficulty in discarding items (11). A study of young adults demonstrated that executive functioning difficulties associated with ADHD were related to hoarding behaviors and hoarding-related beliefs (12), suggesting that. ADHD-related cognitive dysfunction plays a crucial role in the development and maintenance of hoarding symptoms. Network analyses suggest associations between comorbidities and excessive acquisition behaviors (13). Given that excessive acquisition is a strong predictor of distress and functional impairment in individuals with hoarding symptoms (14), a detailed understanding of the comorbidities associated with HD is clinically important.

However, previous studies have largely relied on self-report measures assess HD and psychiatric comorbidities (7, 8). HD is a diagnostically complex condition that requires excluding other disorders and careful evaluation of the motivations underlying hoarding behaviors (15). Although widely used self-report measures, such as the Saving Inventory-Revised (SI-R) (16), Clutter Image Rating (CIR) (17), and Hoarding Rating Scale (HRS) (18), have demonstrated good psychometric properties, they cannot exclude hoarding behaviors attributable to other conditions, including OCD, autism spectrum disorder(ASD), depressive disorders, psychotic disorders, and dementia. These instruments are valuable for screening purposes, but are insufficient for establishing a definitive diagnosis of HD. Similarly, the semi-structured HRS interview does not fully address this limitation.

The Structured Interview for Hoarding Disorder (SIHD) (19) is a widely used diagnostic interview that assesses the core features of HD, including difficulty discarding possessions, excessive acquisition, clutter in living areas, associated distress or functional impairment, and exclusion criteria confirming that symptoms are not better explained by another psychiatric disorder. The SIHD is a semi-structured assessment tool designed to assist clinicians and trained interviewers in making a diagnosis and has been used extensively in treatment and clinical research studies (20–22).

ADHD was assessed using a semi-structured diagnostic interview. The Conners’ Adult ADHD Diagnostic Interview for Diagnostic and Statistical Manual of Mental Disorders (DSM), Fourth Edition (CAADID) (23) is a DSM-based semi-structured interview that evaluates ADHD symptoms and associated functional impairment from childhood through adulthood. Only a few studies have diagnosed both HD and ADHD using semi-structured interviews rather than self-report measures, and reported inconsistent comorbidity rates (24, 25). Even a large network analysis study that used diagnostic tools such as SIHD and reported ADHD as the condition most strongly associated with HD relied on participants’ self-reported history of ADHD diagnosis rather than a structured diagnostic interview for ADHD (3).

Therefore, a systematic assessment of psychiatric comorbidities, including ADHD, using clinician-administered semi-structured interviews for HD and comorbid disorders is important to provide a more accurate estimate of the frequency of psychiatric comorbidity in individuals with HD.

This study rigorously diagnosed HD and comorbid psychiatric disorders using clinician-administered structured interviews, enabling a more accurate assessment of their relationships. Based on the literature, the following hypothesis was formulated:

*MDD is the* most *common comorbidity in individuals with HD.*

*ADHD, like depression, is frequently* comorbid *with HD.*

## 2 Materials and methods

### 2.1 Participants and procedures

This study included 85 patients who visited the Department of Neuropsychiatry at Kyushu University Hospital to seek treatment for hoarding symptoms. Participants were recruited through three pathways: (1) referrals from the OCD specialty outpatient clinic of the Department of Neuropsychiatry at Kyushu University Hospital; (2) referrals from local general psychiatric clinics; and (3) public advertisements in newspapers. The recruitment period lasted from May 2016 to September 2022.

All participants underwent SIHD to determine a DSM-5 diagnosis of HD. Consequently, 68 individuals were diagnosed with HD. To control for the potential influence of age and comorbid conditions that could affect cognitive function, the exclusion criteria were: (1) younger than 16 years or older than 65 years; (2) current or past 12-month diagnosis of drug or alcohol dependence; and (3) presence of a severe neurological disorder.

Using date from the Brain Health Registry, researchers from the University of California, San Francisco, reported that approximately 10% of individuals with HD have a history of mild cognitive impairment (MCI) (3). Notably, hoarding behaviors associated with dementia ware not diagnosed as HD; however, the diagnostic criteria used in the present study did not allow for a precise diagnosis of MCI, which could have introduced bias. Therefore, participants aged 65 years or older were excluded to minimize the potential effects of age-related neurodegenerative diseases and maintain the focus of the study on the association between HD and ADHD. Patients with substance use disorders, including alcohol use disorder, were also excluded. Although substance use, particularly alcohol, is not associated with hoarding behavior (26), substance use disorders can lead to cognitive decline and functional impairment (27), which may mimic or exacerbate hoarding symptoms. Excluding these participants ensured that any observed associations between HD and psychiatric disorders were not attributable to secondary effects of substance use.

Diagnostic and interview assessments were conducted by psychiatrists with over 3 years of clinical experience who worked in a specialized outpatient clinic for obsessive–compulsive spectrum disorders. Written informed consent was obtained from all participants before the commencement of the study. The Ethics Committee of Kyushu University Hospital approved this study (Approval No.: 22287-00).

### 2.2 Measures

#### 2.2.1 Structured interview for hoarding disorder

The presence and severity of hoarding symptoms based on DSM-5 diagnostic criteria were assessed, using the SIHD, a clinician-administered diagnostic tool developed for standardized clinical evaluation of HD (19). The questionnaire consisted of items regarding the diagnostic criteria for HD. The SIHD systematically evaluates the core diagnostic features of HD, including difficulty discarding possessions, excessive saving of items, cluttering of living spaces, associated distress or functional impairment, and exclusion criteria to determine whether the symptoms are not attributed to another mental or medical disorder. Insight was assessed using a specific item from the SIHD: “Do you believe that your hoarding behaviors (including difficulty discarding, clutter, or excessive acquisition) are problematic to any degree?” Participants were classified as having good insight if they acknowledged that their hoarding-related beliefs and behaviors were problematic. In contrast, participants who were strongly convinced that their hoarding-related beliefs and behaviors were not problematic, despite clear contrary evidence, were classified as having poor insight to the contrary.

The SIHD has demonstrated strong psychometric properties. Specifically, it shows excellent inter-rater reliability for diagnostic items (Kappa (κ) = 0.87) and good convergent validity with self-report measures of hoarding, such as the HRS–Self Report(HRS-SR). In this study, we used the Japanese translation of the SIHD with permission from the developer. Notably, the validity of the Japanese version has not yet been evaluated in any published literature.

#### 2.2.2 Conners’ adult ADHD diagnostic interview for DSM-IV

CAADID was administered to assess ADHD (23). The CAADID is a semi-structured interview designed to evaluate adult ADHD based on DSM-IV criteria. It elicits symptoms of inattention and hyperactivity/impulsivity separately for adulthood and childhood. The evaluator made a diagnostic judgment based on the degree of functional impairment caused by the symptoms in domains such as self-concept, academic/work performance, home life, and interpersonal relationships. Based on the symptom profile, individuals are classified into inattentive, hyperactive-impulsive, or combined types. ADHD was diagnosed only when functional impairments were evident in both childhood and adulthood.

This interview has demonstrated adequate psychometric properties. Regarding test–retest reliability, the κ coefficients for individual symptoms reportedly fall within the fair to good range, while the κ coefficient for the overall diagnosis was 0.67 and 0.69 for adult ADHD and retrospective childhood diagnosis, respectively. These values indicate at least moderate to substantial agreement, supporting its validity as a diagnostic tool.

#### 2.2.3 Structured clinical interview for DSM

The Structured Clinical Interview for DSM-IV Axis I Disorders (SCID) is a structured interview for diagnosing DSM-IV Axis I disorders and comorbid psychiatric conditions (28). Following the publication of the Japanese version of the DSM-5, SCID-5 has been used since February 2021 (29). The SCID-5 is a semi-structured diagnostic interview designed to systematically assess major psychiatric disorders based on the DSM-5 criteria, and is widely used in both clinical and research settings.

The SCID-5 has demonstrated good psychometric properties. In joint interview settings, the rate of positive agreement exceeded 75% for all diagnoses except schizophreniform disorder, and the rate of negative agreement exceeded 98% across diagnoses. The coefficients of diagnostic agreement were generally excellent, with most diagnoses yielding values > 0.75. In test-retest evaluations of interrater reliability, the agreement rates were slightly lower but remained within acceptable limits. The negative agreement rate remained above 92% for all diagnostic categories, and κ values were excellent (≥0.75) for bipolar disorder, MDD, mood disorders, and psychotic disorders.

#### 2.2.4 Clutter image rating

CIR is a visual assessment tool developed to evaluate the severity of “clutter” associated with HD (17). This scale consists of nine photographs showing the degree of clutter in three representative rooms in the home: the kitchen, living room, and bedroom. Participants selected the photograph that most closely resembles the condition of each room in their home, and the CIR score was calculated by averaging the evaluation values across the three rooms.

Psychometric properties were evaluated in a validation study, and internal consistency, test–retest reliability, and interrater reliability were found to be good. Convergent validity with other hoarding assessment scales using interviews or questionnaires was also good. We used a validated Japanese translation of the instrument (30).

#### 2.2.5 Saving inventory-revised

The SI-R is a self-report questionnaire that assesses the severity of hoarding in three domains: difficulty discarding, clutter, and excessive acquisition (16). This questionnaire was developed based on the conceptual framework of compulsive hoarding proposed by Frost and Hartl, which identifies three core elements that prevent the normal use of living spaces: excessive acquisition, difficulty discarding, and clutter. To comprehensively capture these dimensions and reflect the associated distress and functional impairment, items were created from earlier versions of the scale and from pilot testing. Each item is rated on a five-point scale ranging from 0 to 4, with total scores ranging from 0 to 92. In the present study, the SI-R data were missing for nine participants.

The SI-R demonstrates good psychometric properties, including high internal consistency, test–retest reliability, and convergent validity. This scale has effectively distinguishes individuals with HD from non-hoarding controls and correlates strongly with other measures of clutter and hoarding-related impairment. In this study, we used a validated Japanese translation of the instrument (31).

#### 2.2.6 Hoarding rating scale–self report

To assess the severity of hoarding symptoms, we used the self-report version of the Hoarding Rating Scale-Interview (HRS-I) (18), the HRS-SR (32). Similar to the interview format, the HRS-SR consists of five items: difficulty using living spaces due to clutter, difficulty discarding possessions, excessive acquisition of possessions, and psychological distress. Each item is rated on a nine-point scale ranging from 0 (no impairment) to 8 (extreme difficulty), with total scores ranging from 0 to 40.

The HRS-I demonstrated high internal consistency and interrater reliability, strong correlations with other hoarding measures, and the ability to accurately discriminate between individuals with and without HD. The HRS-SR was highly correlated with the HRS-I (r = .74–.92), with an agreement rate of 73% between self-reported and interviewer-rated diagnostic statuses. In this study (18), we used a validated Japanese translation of the instrument (33).

### 2.3 Statistical analysis

Statistical analyses were conducted using IBM Statistical Package for Social Sciencesversion 29.0.0.0 (241).

Exploratory analyses were performed to examine whether hoarding severity differed according to the presence of psychiatric comorbidities, the number of psychiatric comorbidities, ADHD, or current MDD. Appropriate parametric or non-parametric tests were used according to data distribution, and effect sizes were calculated for all comparisons.

In addition, exploratory, and binary logistic regression analyses were performed to identify factors associated with psychiatric comorbidity. The presence of any psychiatric comorbidity was entered as the dependent variable, and age, sex, years of education, and family history of hoarding behavior were included as independent variables. Model fit was assessed using the Hosmer–Lemeshow goodness-of-fit test.

Data were missing for nine individuals for the SI-R, six for years of education, 10 for family history of hoarding behavior, 10 for excessive acquisition, and eight for insight. Analyses were conducted using available-case analysis, and participants with missing data were excluded only from analyses involving the corresponding variables.

## 3 Results

Of the 85 individuals assessed, 68 (28.7% men and 71.2% women) met the diagnostic criteria for HD (**Table 1**). The age of the participants ranged from 19 to 64 years (Mean, 39.4 years). overall 53 (80.3%) had at least one comorbid psychiatric disorder, whereas 13 (20%) were diagnosed with HD only.

**Table 1:** Clinical characteristics and comorbidities of the patients.

Variable	Hoarding disorder (n = 68)	95% CI, Lower	95% CI, Upper
Demographic and Clinical Characteristics			
Sex male/female	20/48	-	-
Age, years, mean(SD)	39.81(12.48)	-	-
family history of hoarding behavior +/-	29/29	-	-
Years of Education, mean (SD)	13.84(2.41)	-	-
SIR total, mean (SD)	61.51(14.65)	-	-
HRS-I total, mean (SD)	24.85(8.49)	-	-
CIR total, mean (SD)	12.22(5.03)	-	-
CIR bedroom, mean (SD)	4.28(1.91)	-	-
CIR kitchen, mean (SD)	3.75(1.84)	-	-
CIR living, mean (SD)	4.19(2.05)	-	-
Excessive acquisition +/-	52/6	-	-
Insightpoor/good	10/50	-	-
ADHD	20(29.4%)	18.6	40.3
MDD	14(20.95%)	11.2	30.7
OCD	18(26.47%)	16.0	36.9
Specific phobia	8(11.76%)	4.1	19.4
Social anxiety disorder	7(10.29%)	3.1	17.5
Panic disorder	4(5.88%)	0.3	11.5
Posttraumatic stress disorder	4(5.88%)	0.3	11.5
Psychotic disorder	3(4.41%)	0.0	9.3
Agoraphobia	2(2.94%)	0.0	7.0
GAD	2(2.94%)	0.0	7.0
Somatic symptom disorder	1(1.47%)	0.0	4.3
Bulimia nervosa	1(1.47%)	0.0	4.3

SD, standard deviation; SI-R, Saving Inventory-Revised; HRS-I, Hoarding Rating Scale-Interview; CIR, Clutter Image Rating; ADHD, Attention deficit/ hyperactivity disorder; MDD, Major depressive disorder; OCD, Obsessive-compulsive disorder; GAD, Generalized anxiety disorder. Data were missing for nine individuals for the SI-R, six for years of education, 10 for family history of hoarding behavior, 10 for excessive acquisition, and eight for insight. Some participants had multiple psychiatric diagnoses.

Psychiatric comorbidities were assessed using the SCID. The most common comorbid disorder was ADHD, diagnosed in 19 participants (29%). Among these, 55% and 45% met the criteria for the predominantly inattentive subtype, and the combined type, respectively. No participant met the criteria for the hyperactive-impulsive subtype respectively.

OCD and MDD ware diagnosed in 17(26%) and 14(21%) participants, respectively. Regarding anxiety disorders, the comorbidity rates for anxiety disorders were: social anxiety disorder (12%); specific phobia (12%), panic disorder (6%), agoraphobia (3%), and generalized anxiety disorder (3%).

Exploratory analyses showed that participants with at least one psychiatric comorbidity had significantly higher SI-R scores than those without psychiatric comorbidity. In contrast no significant differences were observed in HRS-SR or CIR scores. Hoarding severity did not differ significantly according to the number of psychiatric comorbidities (0, 1, 2, or ≥3) on the SI-R, HRS-SR, or CIR scales (all *p* > 0.05). The SI-R did not reach statistical significance; however it showed a relatively larger effect size than the other severity measures (*H* = 7.390, *df* = 3, *p* = 0.060, η²H = 0.068). Furthermore, no significant differences in SI-R, HRS-SR, or CIR scores were observed between participants with and without ADHD or between those with and without current MDD.

In an exploratory binary logistic regression analysis, none of the examined variables (age, sex, years of education, or family history of hoarding behavior) were significantly associated with the presence of psychiatric comorbidities (all *p* > 0.05). The model demonstrated adequate fit according to the Hosmer–Lemeshow test (*p* = 0.838), and the Nagelkerke R² was 0.101.

## 4 Discussion

In this study, HD and psychiatric comorbidities were rigorously assessed using clinician-administered structured interviews to clarify the clinical characteristics of HD. Contrary to our hypothesis and previous studies identifying MDD as the most common psychiatric comorbidity, ADHD was the most prevalent comorbid disorder in our sample. In contrast, the prevalence of current MDD and GAD was relatively low. These findings indicate that persistent neurodevelopmental conditions and episodic psychiatric disorders may have different clinical relevance in individuals with HD. However, given this study’s cross-sectional design, no conclusions can be drawn regarding their temporal, causal, or mechanistic relationships with HD. Nevertheless, our findings provide a potentially useful perspective for understanding the psychiatric comorbidity profile of HD. The relationships between HD and its major psychiatric comorbidities are discussed below.

### 4.1 HD and ADHD

ADHD was the most common comorbidity (29%) in this study. The use of clinician-administered structured interviews enabled accurate ADHD diagnosis. Prior research using self-reported measures also found higher rates of ADHD in individuals with HD than in those with OCD (1), and our findings using clinician-administered structured interviews were consistent with these results.

Neuropsychological studies on HD have yielded inconsistent findings; however, because laboratory-based tasks assess only specific aspects of attention under restricted conditions (34–36), multiple studies suggest that ADHD-related attentional deficits may exacerbate difficulties in organization, decision-making, and procrastination behaviors observed in HD (37, 38). Consistent with this, Morein-Zamir et al. (2021) reported that inattention predicts the severity of hoarding (7). These findings suggest that ADHD is not merely an incidental comorbidity of HD but may share cognitive vulnerabilities involving attention and executive functioning. Such cognitive difficulties may interfere with sorting, organizing, and discarding possessions and may contribute to the development or maintenance of hoarding behaviors.

The predominance of ADHD rather than MDD in this present study may also reflect differences in the clinical course of these disorders. ADHD is a neurodevelopmental disorder with onset in childhood. Symptoms may become less prominent in some individuals during adulthood; however, difficulties in attention and executive functioning often persist over time. In contrast, MDD is generally episodic, and individuals may no longer meet the diagnostic criteria remission or treatment. Consequently, structured interviews assessing current diagnoses may have been more likely to identify persistent ADHD symptoms and related functional impairments than past depressive episodes that had already improved.

A possible clinical hypothesis is that attentional and executive-function difficulties associated with ADHD may contribute to the development or maintenance of hoarding behaviors. As HD becomes chronic, the resulting functional impairment, social isolation, family conflict, and psychological distress may increase vulnerability to depressive episodes. Within this framework, ADHD may be closely associated with the cognitive vulnerabilities underlying HD, whereas MDD may occur in some patients along with the burden of chronic hoarding. However, this interpretation remains hypothetical, and the cross-sectional design of this study does not allow drawing conclusions regarding temporal sequence, causality, or underlying mechanisms. Nevertheless, this framework may help explain why ADHD was the most common comorbidity despite the high lifetime frequency of depressive episodes.

As difficulties in attention and executive functioning may affect the organization and discarding of possessions, ADHD represents a clinically important comorbidity that should be assessed in patients with HD. Furthermore, a recent study of adults with ADHD reported improvements in hoarding symptoms, particularly clutter and excessive acquisition, along with improvements in ADHD symptoms following methylphenidate treatment (39). In this study, we did not directly examine patients with HD. Therefore, the findings should be interpreted cautiously, as it raises the possibility that identifying and treating comorbid ADHD may influence attentional dysfunction and certain some aspects of hoarding symptomatology. Accordingly, our findings highlight the importance of systematically assessing childhood-onset attentional symptoms and current ADHD-related functional impairments during the clinical evaluation of HD. Future longitudinal studies should examine the utility of routine ADHD screening in HD and clarify how comorbid ADHD is associated with treatment selection, treatment response, and long-term outcomes.

### 4.2 HD and MDD

Fourteen participants (21%) met the diagnostic criteria for MDD, a prevalence rate lower than those reported in previous studies, which found comorbidity rates of 32.8% - 56.7% and identified MDD as one of the most common comorbid conditions in HD (1, 24, 25, 40). Thus, our findings differed from those of earlier studies. A previous comparison of patients with OCD with and without hoarding symptoms showed that the group with hoarding symptoms had a higher rate of depression (42.9% vs. 21.9%) (41). Moreover, previous literature suggests a positive association between hoarding severity and depressive symptoms (42).

A possible explanation for the relatively low comorbidity rate of MDD observed in the present study is a selection bias. Previous studies recruited participants through multiple pathways to obtain representative samples of individuals with HD, including those who might not seek treatment (1, 24, 40). However, these studies also included participants referred from mental health clinics. Therefore, they may have included individuals who had already been connected to psychiatric services for depression treatment or other psychiatric conditions. The proportions of recruitment sources were not described in sufficient detail to draw definitive conclusions; however, the comorbidity rate of MDD in patients undergoing HD may have been overestimated in previous studies because of the inclusion of medically engaged samples.

In contrast, this study’s participants mainly sought treatment for hoarding symptoms. When depressive symptoms are more severe, treatment for depression may be prioritized, and intervention for hoarding behaviors may be postponed. Therefore, depressive symptoms in participants who voluntarily sought treatment for hoarding symptoms may already have been relatively well controlled. In this respect, this study’s findings may better reflect the psychiatric comorbidity profile of patients with HD encountered in routine clinical practice.

The episodic nature of MDD should also considered. Although 21% of participants met the criteria for current MDD, 31 individuals (47%) reported a lifetime history of a major depressive episode. This suggests that depressive symptoms may occur frequently over the clinical course of HD, even when patients do not meet the criteria for an active depressive episode at the time of assessment. The discrepancy between the relatively low prevalence of current MDD and the high frequency of past depressive episodes may partly explain why ADHD, a more persistent condition, emerged as the most common current psychiatric comorbidity in this study.

Notably, relatively younger HD cohorts with a mean age below 50 years have shown high depression rates of 50% - 56%, whereas lower rates of 32% - 33% have been reported among older patients with HD aged over 50 years. A possible explanation is that strong attachment to possessions in severe hoarding may provide a sense of safety through acquisition and possession, reducing social isolation and emotional distress (42). The sense of reassurance obtained from clutter may buffer emotional distress and, consequently, lower the rate of current depression. HD tends to worsen chronically over time (40); hence increasing hoarding severity with age could be associated with a lower comorbidity rate of depression. However, in this study, patients with HD were relatively young, with a mean age of 39 years, yet their depression rate was lower than those reported in previous studies. Therefore, age differences alone are unlikely to fully explain the discrepancy observed in the present study.

Collectively, the relatively low prevalence of current MDD observed in this study may be attributable to multiple factors, including the exclusion criteria, the recruitment of patients who primarily sought treatment for hoarding symptoms, prior treatment or remission of depressive symptoms, and the episodic nature of MDD. In contrast, the frequency of past depressive episodes was high, suggesting that depression is a common clinical feature of HD. Accordingly, in addition to current psychiatric diagnoses, the assessment of lifetime depressive history may be important in the clinical evaluation of patients with HD.

### 4.3 HD and OCD

Previous studies have reported comorbidity rates of OCD in patients with HD ranging from 6.1% to 25.7% (1, 18, 24, 25, 43). Although we anticipated a relatively low rate, the observed rate was slightly higher (26%). This may be attributable to the participant recruitment at our hospital’s outpatient clinic. Nonetheless, the similarity between our findings and those of previous studies supports the conceptual distinction of HD and OCD as separate disorders.

### 4.4 HD and GAD

Reportedly, anxiety disorders, particularly social anxiety and generalized anxiety disorders (GAD), co-occur with HD. Notably, significant hoarding symptoms have been observed in 12–25% of patients with specific phobias or other anxiety disorders (44). Previous studies have also reported GAD comorbidity rates ranging from 0% to 24.4% (1, 24, 25, 43). A recent network analysis found no direct relationship between hoarding symptoms and GAD, despite the high self-reported prevalence of GAD (>30%) among individuals with HD (3). This study found that 72% of patients on HD with GAD also had comorbid depression, suggesting that the high prevalence of GAD may be driven by depression. In our study, the low rate of depression may explain the low GAD comorbidities.

### 4.5 Limitations

This study had some limitations. First, ASD was not assessed because it was not included in the SCID evaluation; therefore, we could not determine the prevalence of ASD comorbidity, which has been suggested to be associated with HD.

Second, excluding individuals younger than 16 years or older than 65 years, and those with comorbid substance use disorders may have limited the generalizability of our findings. The mean age of the study participants was 39.4 years, whereas the mean age reported in HD research samples generally exceeded 49 years (45). We believe that our exclusion criteria contributed to the relatively younger age of our sample. In addition, these exclusion criteria may also have influenced the observed prevalence of psychiatric comorbidities, including MDD.

Third, most participants were female. Because several psychiatric disorders, including MDD and anxiety disorders, are generally more prevalent among women, the sex distribution may have affected the observed prevalence of psychiatric comorbidities. However, despite the predominance of female participants, the prevalence of current MDD was lower than that reported in previous studies, suggesting that differences in sex distribution alone are unlikely to fully explain our findings.

Fourth, participants were recruited from specialized outpatient clinics at Kyushu University Hospital and affiliated institutions. Consequently, many patients may have sought treatment not only for HD but also for other psychiatric conditions, raising the possibility of referral bias. In particular, the relatively high prevalence of OCD observed in this study may have been influenced by the recruitment pathway.

Fifth, our sample was limited to individuals seeking treatment, which may restrict the generalizability of our findings to a broader population of individuals with HD, including those who do not seek clinical care. Sixth, the Japanese version of the SIHD has not undergone formal psychometric validation. Although the SIHD is a structured diagnostic interview based on DSM-5 criteria and assessments were conducted by clinicians experienced evaluating of HD, the reliability and validity of the Japanese version are yet to be established. Therefore, a certain degree of diagnostic misclassification cannot be excluded. Future studies should formally evaluate the reliability and validity of the Japanese SIHD and confirm the reproducibility of the present findings using validated diagnostic instruments.

Finally, psychiatric diagnoses were assessed using SCID-IV and SCID-5 during the study period. Diagnoses were established by experienced clinicians; however diagnostic consistency between the two versions was not formally evaluated. Therefore, some degree of diagnostic variability attributable to differences between the DSM versions cannot be excluded.

Additional exploratory analyses suggest that psychiatric comorbidity may be associated with greater self-reported hoarding symptoms. However, given the exploratory nature of these analyses and the limited sample size, the findings should be interpreted with caution and replication in larger samples is required.

No significant demographic or family-history predictors of psychiatric comorbidity were identified through exploratory logistic regression analysis. Although the overall model explained approximately 10% of the variance in comorbidity status, the findings suggest that factors other than age, sex, education, and family history may play a greater role in determining psychiatric comorbidity among individuals with HD.

## 5 Conclusions

In this study, we examined patterns of psychiatric comorbidity in patients with HD using structured clinical interviews. ADHD was the most common current psychiatric comorbidity. A minor strength of this study was the use of a clinician-administered diagnostic interview for ADHD, an approach rarely used in HD research. However, given the cross-sectional design, no conclusions can be drawn regarding causal or mechanistic relationships between ADHD and HD. Larger longitudinal studies are needed to clarify the clinical significance of psychiatric comorbidity, including ADHD, and its associations with treatment response and long-term outcomes in patients with HD.

## Data Availability

The raw data supporting the conclusions of this article will be made available by the authors, without undue reservation.
